# Effect of whole-day work on surgical performance during simulated laparoscopic surgery: study protocol for a controlled cross over laboratory trial

**DOI:** 10.3389/fpubh.2024.1423366

**Published:** 2024-11-14

**Authors:** Anaïs Sevestre, Robin Souron, Thibault Deschamps, Aurélie Sarcher, Thibault Thubert, Vincent Dochez

**Affiliations:** ^1^Nantes Université, CHU Nantes, Service de Gynécologie-Obstétrique, Mouvement - Interactions - Performance, Nantes, France; ^2^Nantes Université, Mouvement - Interactions - Performance, Nantes, France

**Keywords:** performance, surgery, laparoscopy, working day, training, fatigue

## Abstract

**Introduction:**

Laparoscopy has become a fundamental aspect of surgery, presenting new challenges such as fatigue, encompassing both muscular and cognitive components. Given its potential to affect surgical precision and create difficulties for the surgeon, it is crucial to study the mechanisms of fatigue for patient safety and the well-being of surgeons. This study aims to demonstrate the influence of general fatigue on surgeons’ performance, incorporating assessments of movement quality through balance, kinematics, and muscle activation, as well as perceived workload. Additionally, the study seeks to evaluate how surgeons’ experience may affect fatigue outcomes.

**Methods and analysis:**

A controlled cross-over laboratory trial involving 29 residents and surgeons from the obstetrics and gynecology department of Nantes University Hospital is underway. Recruitment started in March 2023 and ended in September 2023. Participants with varying levels of experience perform two one-hour sessions of training box exercises, one in the morning (control condition) and the other at the end of a workday. The primary outcome is a composite score derived from the time to complete the Suturing and Knot Tying Training and Testing (SUTT) exercise, along with the number and quality of stitches. Secondary outcomes include perceived fatigue, discomfort, physical strain, muscle tension, mental workload, muscle activation (measured by surface electromyography), balance (measured using a force platform), and kinematics (measured using motion capture).

**Ethics and dissemination:**

The study received ethical approval from the local ethics committee CERNI in December 2022 (n°13,122,022). Results will be presented in international conferences, submitted to peer-reviewed journals, and serve as a feasibility study for subsequent publications.

## Introduction

### Background

Minimally invasive surgery has become commonplace in contemporary surgical practice, due to benefits for patient outcomes, including reduced hospital stay, decreased postoperative morbidity and alleviation of pain (1, 2). However, this evolution has also introduced ergonomics challenges, leading to an increased incidence of injuries across all surgical specialties (3). The prevalence of musculoskeletal disorders among minimal invasive surgeons is estimated to range from 22 to 74% (4). Among these specialists, gynecological surgeons seem to be particularly vulnerable to work-related musculoskeletal disorders with prevalence rates from 73 to 100% (5). Specifically in gynecologic surgery, the lateral position of surgeons working in the pelvis is known to be physically and cognitively demanding, although poorly studied (6). It is known that poor ergonomics can increase workload and impact surgical performance or induce surgical injuries (7). The requirements of maintaining static and specific postures, performing repetitive movements and the use of specific and constraining laparoscopic instruments may favor the apparition of chronic musculoskeletal disorders. This phenomenon is significantly increased by muscular fatigue and strain during and after prolonged laparoscopic procedure (8, 9). Cognitive fatigue may also develop during prolonged laparoscopic procedures. This kind of fatigue is multifaceted, originating from high concentration demands, complex task execution, and time-sensitive decision-making pressures inducing intraoperative stress (10–12). The interaction between these two fatigue dimensions can help to describe the overall workload (13). All these specificities related to laparoscopy encourage consideration of the impact of a day’s work and repeated laparoscopies on the performance of surgeons, specifically in gynecology.

The impact of fatigue on laparoscopic performance is not well documented. It is understood that repetitive exercises and practice may induce fatigue, as assessed by electroencephalography, and could result in increased errors in laparoscopic simulators (14). Additionally, muscle endurance training might enhance laparoscopic performance, indicating that ergonomic factors and muscle strain could negatively affect performance (15). However, some studies have not demonstrated a statistically significant association between performance and sleep deprivation. This suggests that surgeons may learn to adapt to fatigue, but the coping mechanisms employed could potentially lead to a decrease in movement quality and ergonomics, especially among inexperienced residents (16–18).

Assessment tools that objectively detect a deteriorated movement due to muscular and/or cognitive fatigue among laparoscopic surgeons are not as commonly used as subjective measures (19, 20). Among those subjective measures, muscular fatigue is usually assessed by self-evaluation on visual analogic scale (VAS). Regarding the mental workload induced by repetitive laparoscopic procedures, the National Aeronautics and Space Administration Task Load Index (NASA-TLX) questionnaire is the most commonly used but not always adapted to surgical settings (21, 22). Important intraoperative stressors are not included in the NASA-TLX tool such as distractions or task complexity known to be majors stressors that might impact the perceived workload during surgery (23, 24).

Moreover, we know that fatigue can lead to vestibular disorders in an individual and affect balance. In the context of surgery or simply in the study of ergonomic risks, postural asymmetry emerges as one of the most significant risk factors (25, 26). The assessment of postural asymmetries or fatigue is possible through objective methods such as differences in muscle activation measured by surface electromyography (sEMG). This technique has been extensively used in laparoscopy settings as an objective measure of muscular fatigue (27–29), although it may not always be reliable in detecting fatigue (30, 31). Postural balance can be assessed using force platforms. Force platforms can capture deteriorated postural balance, or instability that may be indicative of fatigue. Concurrently, kinematics can be assessed using motion capture. Kinematic studies can offer insights into movement quality and changes in movement patterns induced by minimally invasive surgery-related fatigue. In previous kinematics studies among laparoscopic surgeons, they seem to experience extended periods of neck rotation and asymmetrical position in shoulders that could justify the prevalence of musculoskeletal disorders among them (32, 33).

Ergonomics and fatigue prevention during minimally invasive surgery should be a concern for surgeon trainees, as they seem to be more exposed, compared to experimented surgeons, to muscle workload and awkward postures that could stick for the rest of their career. They seem to experience greater fatigue suspectedly induced by less adapted postures such as a greater upper body flexion angle or greater shoulders extensions (34, 35). Higher muscle activity is found in unexperienced surgeons especially in lower back muscle and biceps.

The advent of simulation in surgical training offers an unprecedented opportunity to investigate these elements in a controlled, risk-free environment. Pelvic trainer studies usually use the Fundamentals of Laparoscopic Surgery (FLS) task known to be an efficient assessment method to discriminate trainees on their manual skills (36). However, FLS is manly used among general surgery residents compared to obstetrics and gynecology residents (93% vs. 6.2% respectively). Gynecological Endoscopic Surgical Education and Assessment diploma directed by The European Society for Gynaecological Endoscopy and the European Board and College of Obstetrics and Gynaecology seems to be more appropriate to our study since it has been validated with gynecologist surgeons and residents (37).

### The study aims

The primary aim of our study is to assess the impact of a workday on simulation performance and to understand the neuromechanical mechanisms involved if we observe a decline in performance. We will evaluate our ability to detect indirect signs of physical and mental fatigue among gynecologist surgeons in simulation settings.

Secondly, we will do a subgroup analysis to evaluate if surgeon’s experience leads to differences in fatigue-related outcomes.

## Methods and analysis

### Study design

We will compare the fatigue outcomes during 1 h of laparoscopic box test training in the morning with the same session performed after a whole-day work. Our study will be based on a cross over model, each participant will be his own control. We will try to show if the fatigue induced by a full day of work leads to changes in surgeon’s performance, quality of movement including upper body posture, perceived workload, and surgeon’s motivation.

**Figure 1:**
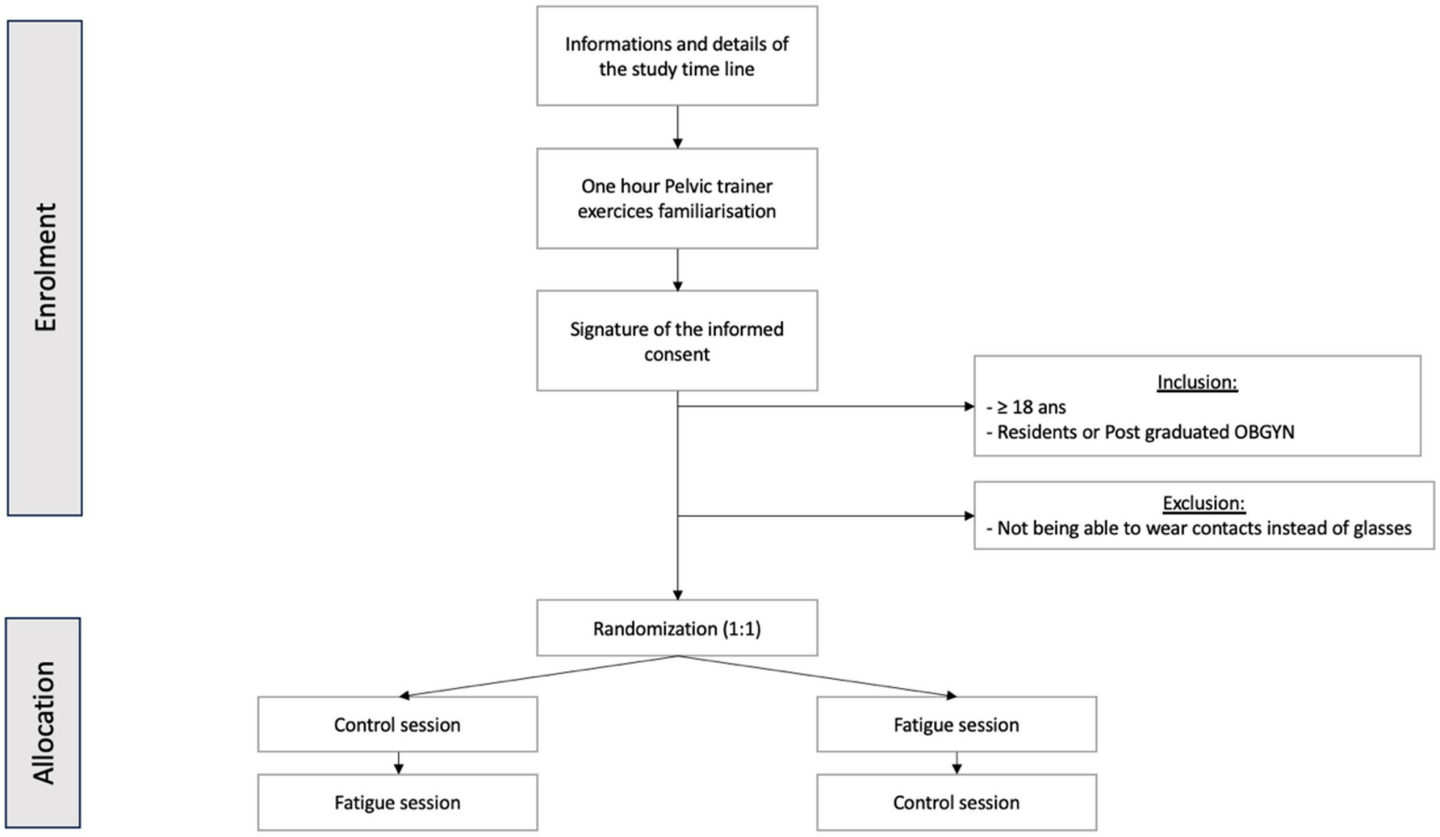
Trial design; obstetrics and gynecology (OBGYN).

We will conduct a controlled cross over study, following the SPIRIT guidelines. Every participant will perform two separate experimental days: (i) the first one planned in the morning, i.e., without any work intervention before testing (control day), (ii) the second one planned at the end of a full workday (fatigue day). The experimental sessions will always be performed on different days. The order of the days will be randomized to control which participant completes the control day or fatigue day first. Each participant will be included for a period of 6 months. Approximately 6 months of recruitment will be required to reach the target sample size. 6 month is the time needing to collect participant’s information, to perform a test training session and randomized participant for the experimental days. Recruitment started in March 2023 and ended in September 2023. Data collection is planned to last until May 2024 and analysis until September 2024. The flow chart of our study is presented in Figure 1.

### Participants

To be eligible for the study, participants must be adult (i.e., over 18 years old), men or women, residents or senior in obstetrics and gynecology (OBGYN). Participants will not be included if they are in guardianship or curatorship, they have major cognitive disorders (e.g., intellectual disability, severe brain trauma), or severe motor or sensory disorders.

Participants will be recruited through direct contact within the gynecology department at the Nantes University Hospital. Clinicians will give a general explanation of the study to potentially eligible participants, along with written information. Participants will be free to ask any questions before signing a written informed consent form (Supplementary material 1). The characteristics of the participants will be collected, including age, weight, height, gender, dominant hand (right or left), number of residency’s semesters or post-residency exercise years, previous Pelvic trainer training, number of hours of sports practice per week, list of sports activities practiced and exposition to unusually activities such as video games (38).

### Experimental procedures

Our experiment will consist in simulated laparoscopy tasks conducted in a Pelvic Trainer (Szabo, ID Trust Medical, Belgium). The control and fatigue sessions will begin by filling out questionnaires (Fatigue sensation scale, physical strain, and motivation). Before starting the simulated experimental task, participants will be equipped with sEMG electrodes and kinematics markers.

We will use an optic in the Pelvic trainer, including a camera and a light source (Optic 0°, Karl Storz). The optic will be connected to a screen in front of the participants (TELE PACK, Karl Storz). The Pelvic trainer will be disposed on a manually adjustable table to adapt the height to each participant according to their preference. Participants will be placed on a force platform (PFA6040M35 Sensix) at the beginning of each set, in a position that will perfectly replicate the one used during standard laparoscopic simulation.

**Figure 2:**
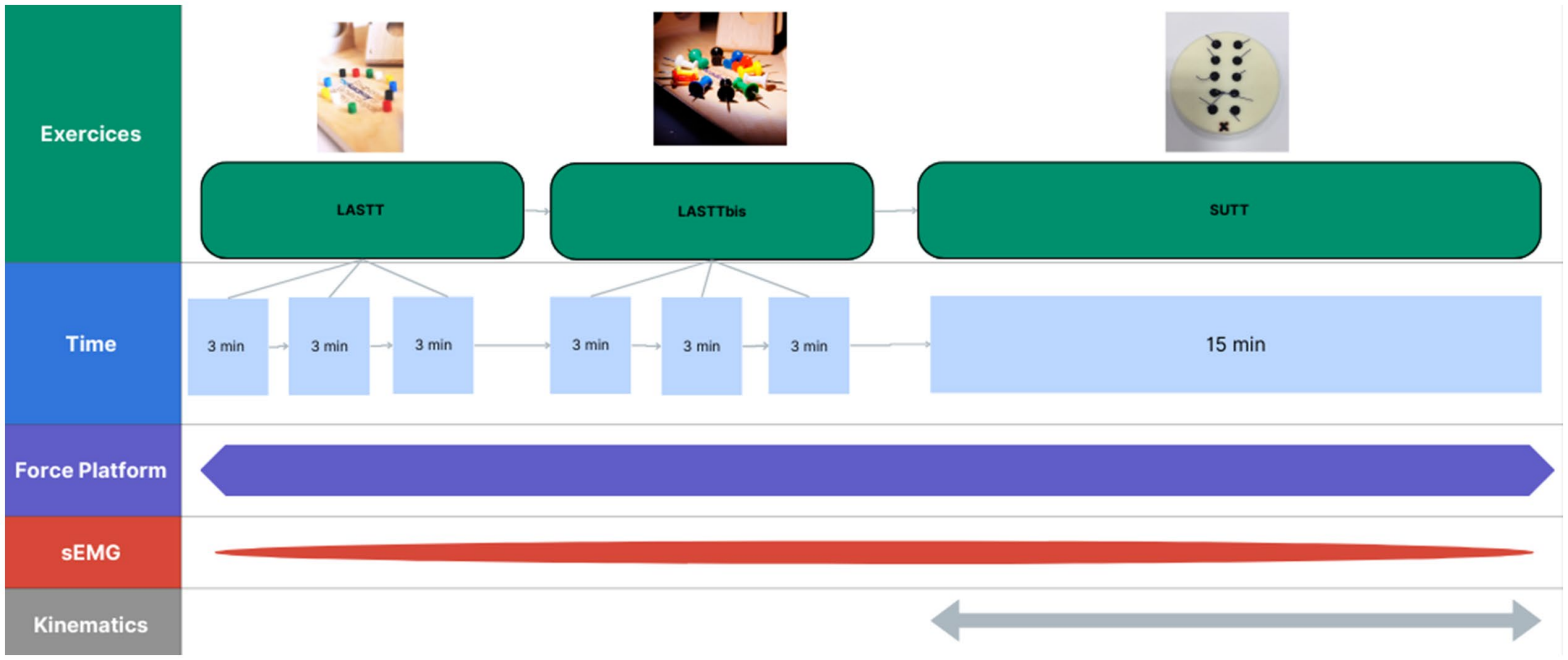
Timeline of an experimental session. LASTT, Laparoscopic Skills Training and Testing; SUTT, Suturing and Knot Tying Training and Testing.

The experimental session will be a series of three different tasks. The Laparoscopic Skills Training and Testing (LASTT) exercises (hand-eye coordination that we will call “LASTT,” and bimanual coordination that we will call “LASTTbis”) will be performed with a 3-min time limit. A 15-min time limit will be set for the Suturing and Knot Tying Training and Testing (SUTT) exercises (see Supplementary material 2 for a detailed description and a graphic representation of each exercise). The Pelvic trainer exercises will be performed on the side of the table to reproduce the conditions of pelvic surgery, a setup that has been underexplored. All tasks will be conducted under the supervision of an experimenter (ASa). EMG, force platform and kinematic data (see below) will be recorded during the experimental session. The timeline of a session is presented in Figure 2.

Participants will be asked to fill out the same questionnaires at the end of the experimental session (see below). Maximal voluntary contractions (MVC) will be performed at the end of each session to obtain sEMG signal that will serve for signal normalization (39). Every MVC exercise will be conducted in an isometric modality of contraction with rigid strap and handles.

### Outcome measurements

Table 1 provides a summary of the measures that will be collected and that will serve for analysis in this study.

**Table 1:** Summary of measures to be collected.

Outcome	Instrument	Time of measurement	Unit
Primary outcome			
Performance	Composite score of time to complete the task associated with quality assessment	SUTT task	0–900
Secondary outcomes			
Muscle activation	RMS static 10th percentile muscle activity	SUTT task	(%MVE)
	RMS Median 50th percentile muscle activity	SUTT task	(%MVE)
	RMS Peak 90th percentile muscle activity	SUTT task	(%MVE)
Muscular fatigue	MPF: Mean power frequency	SUTT task	Hz
Force platform	M/L COP excursion	LASTT, LASTTbis and SUTT task	Cm
	A/P COP excursion	LASTT, LASTTbis and SUTT task	Cm
	M/L COP velocity	LASTT, LASTTbis and SUTT task	Cm/s
	A/P COP velocity	LASTT, LASTTbis and SUTT task	Cm/s
Kinematics	Range of Motion in the 18 DoF		Degree (°)
	Coefficient of multiple correlation (CMC)	SUTT TASK	
Perceived workload	NASA-TLX (English)	Total of 6 items	0–120
	SURG-TLX (English)	Total of 6 items	0–120
Motivation	VAS	Pre-Task and post task	(0–10)
Physical strain	Borg CR-10 Scale: legs, back, neck, Right and left shoulders and right and left arms	Pre and post task	(0–10)
Physical fatigue	VAS	Pre and post test	(0–10)
Effort	VAS	Post test	(0–10)

A/P, Antero posterior; COP, Center of pressure; DoF, Degrees of freedom; M/L, Mediolateral; LASTT, Laparoscopic Skills Training and Testing; MPF, Median power frequency; MVE, maximal voluntary electrical activity; NASA-TLX, National Aeronautics and Space Administration Task Load Index; RMS, Root mean square; SUTT, Suturing and Knot Tying Training and Testing; VAS, Visual analogic scale.

#### Primary outcome: performance during a surgical simulated task

Our primary outcome will be the performance and will be assessed by the amount of time and numbers and quality of the stitches done during the SUTT exercise. We expect a decrease in performance during the “fatigue” session.

The time to complete the task will be assessed (in seconds) for every LASTT, LASTTbis and SUTT exercises combined by a quality assessment of the task (numbers of stiches completed withing 15 min, quality of stiches and nod). Every stich must be done by entering and existing within the black dots. The number of threads passes through each point (between 0 and 10, one pass per point through each black dot). A correct stitch equal one point on a 0–5 scale (5 being the total number of stiches). A correct Knot is rated 2 points if completed correctly and solid. Absence of trauma on the dot is rated on a 0 to 2 scale. All quality scales and the time to complete the task will be added to a online scoring platform called +he Academy’s Online Scoring Platform to produce a composite score going to 0–900.

For data analysis, we will keep only the best time to complete the task among the 3 trials for LASTT and LASTTbis.

#### Questionnaires

##### Fatigue sensation and motivation

Participants will rate their fatigue sensation and task-related motivation using a VAS that ranges from 0 (“no fatigue/no motivation”) to 10 (“extremely fatigued/fully motivated”). They will be asked to rate it before and after the experimental task.

##### Physical strain and muscle tension

Participants will rate their physical strain and muscles tension on the Borg CR-10 Scale (40). Muscle tension refers to the contraction and resistance of muscle during physical activity. The scale ranges from 0 (“no muscle strain”) to 10 (“extremely intense muscle strain”). No muscle tension will be explained as no sensation while 10 represents maximum muscle tension even including cramps or extreme muscle fatigue. Participant were asked to answer for legs, back, neck, right and left shoulders and right and left arms.

##### Mental workload

The mental workload will be assessed at the end of the two experimental sessions with the NASA-TLX questionnaire (21, 22). The NASA-TLX scale consists of six items, i.e., (i) mental demand, (ii) physical demand, (iii) temporal demand, (iv) performance, (v) effort, and (vi) frustration. Participants rated each item on a scale divided into 20 equal intervals anchored by a bipolar descriptor (i.e., high/low). Anchor words for each dimension are available in supplements. This score was multiplied by five, resulting in a final score between 0 and 100 for each item.

We will also ask the participants to fill out a second version of the NASA-TLX adapted to surgery, i.e., the SURG-TLX (23, 41). Dimensions assessed with the SURG-TLX are close but slightly different than the ones assessed with the NASA-TLX: (i) mental demands, (ii) physical demands, (iii) temporal demands, (iv) task complexity, (v) situational stress, and (vi) distraction. This questionnaire has been validated with a good sensibility to surgery specifics stressors in laboratory condition but only with unexperienced surgeons.

##### Muscle activation

Bipolar sEMG (Cometa Miniwave system, Milan, Italy) will be used to measure muscle activation in both the right and left upper trapezius and the anterior deltoid. These muscles have been widely recognized as primary target muscles associated with pain during and after surgery (19, 42, 43). Further, sEMG electrodes will be placed on the biceps brachialis, the short abductor muscle of the thumb, the erector of the spine, and the soleus. The skin will be cleaned with abrasive paste and shaved if there is excessive body hair. Electrodes used will be self-adhesive Ag/AgCl electrodes with an active diameter of 15 mm and inter-electrode distance of 20 mm according to the SENIAM recommendations. Maximal voluntary contractions (MVC) will be performed at the end of each session to obtain sEMG signal that will serve for signal normalization. We chose to measure the MVC at the end of each session rather than at the beginning in order to avoid inducing fatigue for the participants. Participants will be using a rigid strap to perform MVC. Trapezius signals will be recorded with the arms among the body straight and the straps under foot. They will be asked to lift the shoulders in a shrugging motion. Deltoid signals will be recorded with arms at 45° anterior flexion and straps under foot. They will be required to perform an anterior arm elevation with the elbow kept in extension. For Biceps signals participants will be arms among the body in 90° Elbow flexion with a strap under foot, they will be ask to flex the forearm against resistance. Short abductor thumbs will be recorded by grasping a needle holder, as used in SUTT exercises. They will squeeze it in the palm of the hand. Erector spinae were recorded doing a deadlift with resistance coming from strap under feet. Soleus will be assessed in dorsal decubitus with straps underfoot to resist against plantar flexion. Every exercise will be performed for a minimum of 3 s.

The sampling frequency will be 2000 Hz. sEMG analog signals will be analogically integrated into the motion capture software (Nexus 2.12, VICON, Oxford, UK). Analysis of sEMG signals will be done with homemade MATLAB routines (MathWorks, Natick, MA, USA).

The EMG signals from all trials, including MVCs, will be bandpass filtered between 10 and 450 Hz using a zero-lag 4th-order Butterworth filter, followed by rectification. Afterward, they will be low-pass filtered at 9 Hz using a 2nd-order Butterworth filter. For the processing of MVC signals across all muscles and sessions, the highest mean muscle activation value (in mV) will be extracted using a 100 ms sliding window.

From the EMG signals, the Root mean square (RMS) and median power frequency (MPF) will be calculated. The RMS will be expressed as a per cent of the RMS during the MVC, that is, maximal voluntary electrical activity (%MVE). The MPF will be expressed in Hertz (Hz). In all cases, we will evaluate the static, median, and peak level of RMS for each muscle, which can be determined by the 10th, 50th and 90th percentiles of the RMS signal over a specific recording period. For each of the muscles, the average of the 10th, 50th and 90th percentiles of RMS for the SUTT task will be calculated. MPF will be calculated over for the SUTT task (44, 45).

We will conduct a comparison of those data with the “fatigue session” expecting a significative increase %MVE and a significative decrease in MPF.

##### Balance

Force platform data will be collected using Senxis Force Plate Software, and synchronized with muscle activation and kinematics data using an additional analog input. Force platform data will be lowpass filtered at 15 Hz using a 2nd order zero lag Butterworth filter (46). The analysis of force platform-related outcomes will be done using a homemade MATLAB routine. Collected data will be the displacement of the center of pressure (COP), which is the difference between the measurement of the maximum and minimum center of COP in anterior–posterior and medial-lateral. We will also collect the maximum velocities of center of gravity displacement in the two planes: Excursion of COP in anterior–posterior (A/P) and medial-lateral (M/L). Usually, COP excursion are used in ergonomic fields to assess postural outcome in a standing position. We choose to analyze excursion of COP during a task to understand the impact of fatigue on postural during a complex task. We choose to compare COP excursion in A/P and M/L and COP Velocities between the control and “Fatigue session.”

##### Kinematics

**Figure 3:**
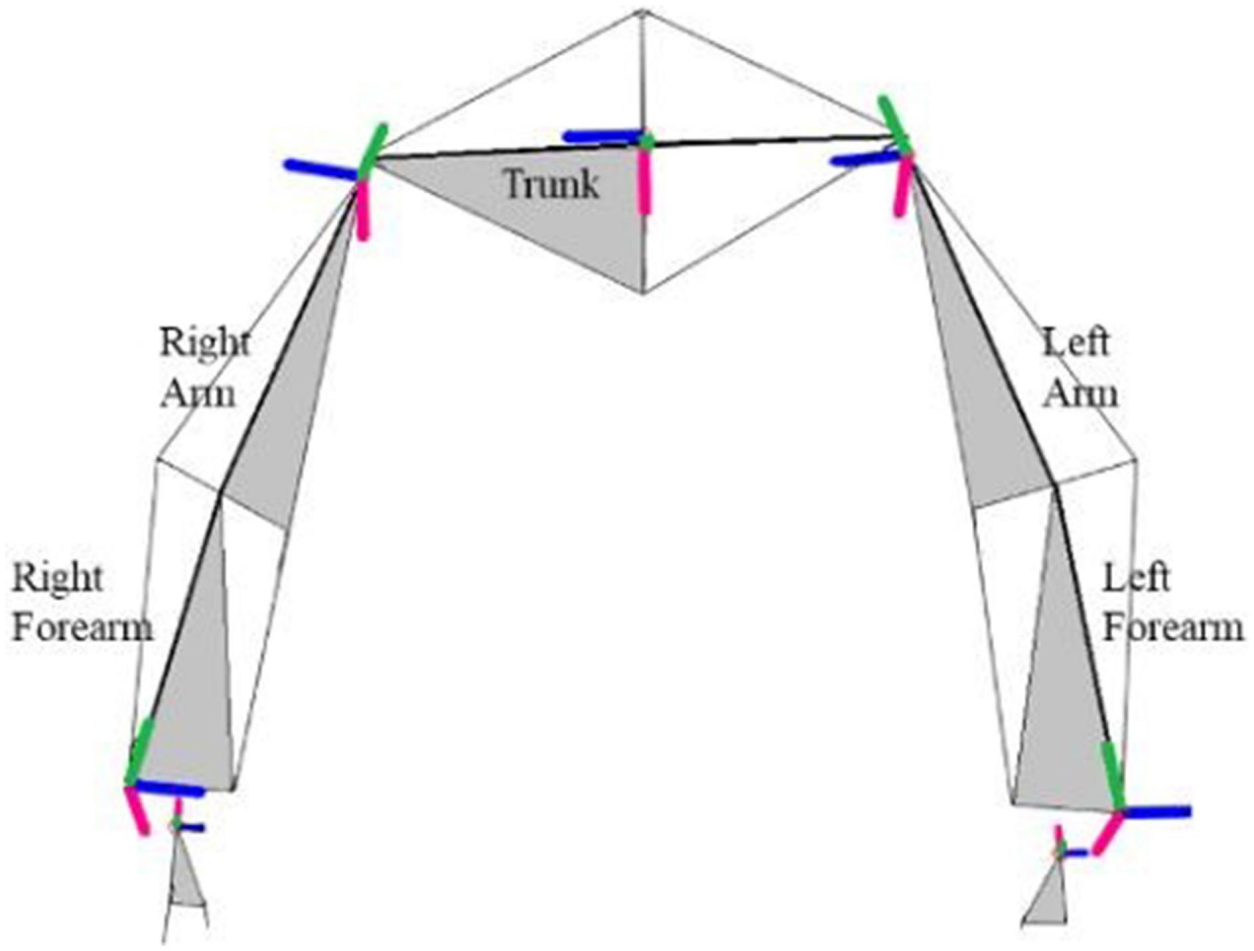
Position and orientation of the segment coordinate system of upper limbs and trunk. Blue axis is for flexion (Z), green is the rotation axis for upper-limb and adduction axis for hand (X), and red is the adduction axis for upper-limb and the axis rotation for hand (Y). In grey the frontal plan from landmarks. Adapted from Maillet et al. (47).

**Figure 4:**
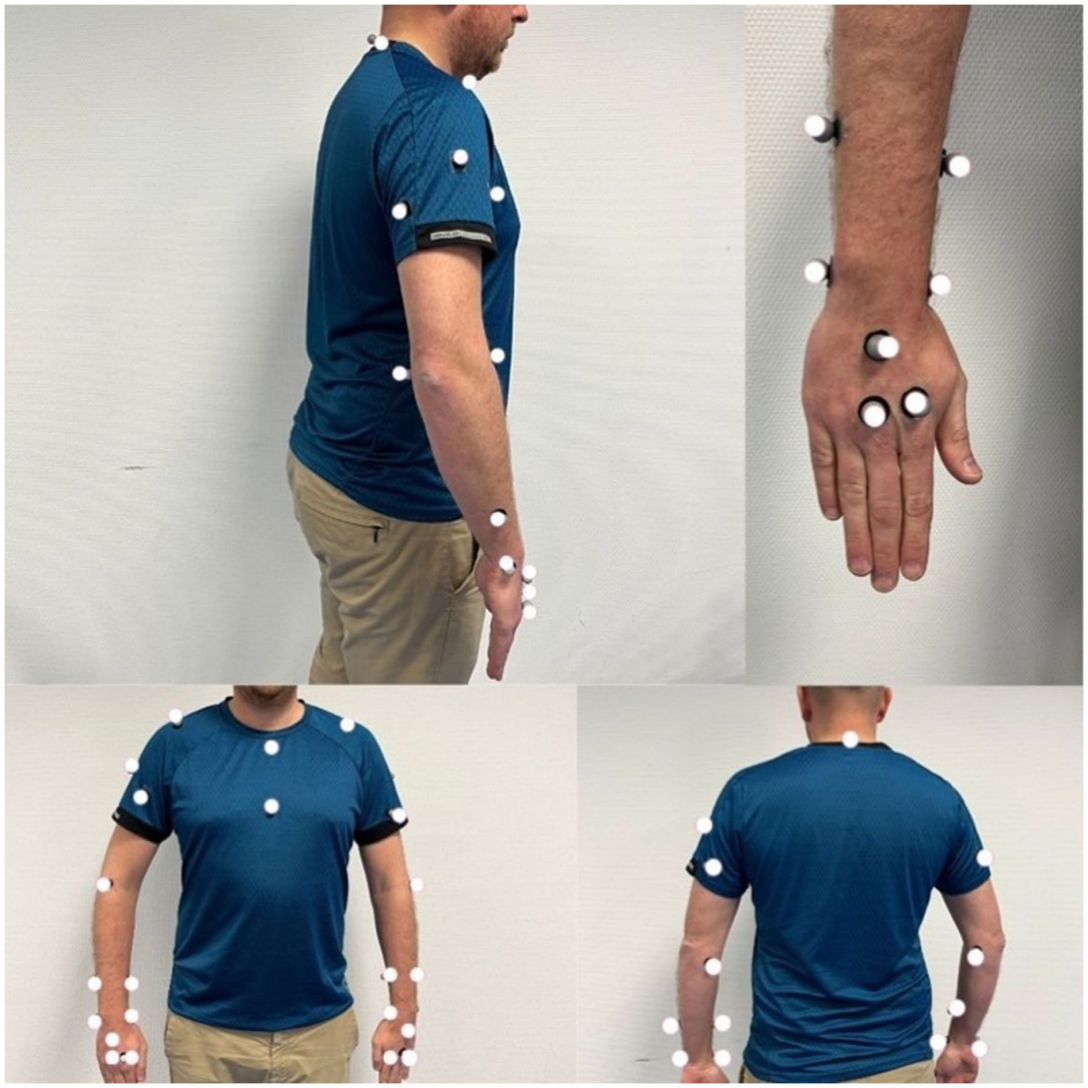
Representation of the placement of kinematics markers.

For kinematics analysis, a motion capture system composed of seven cameras (Vicon Vero 2.2) will be used. We will record kinematics data for seven joints (Figure 3) with a total of 18 degrees of freedom. Three-dimensional position of the thorax, arms, forearms, hands will be considered during the movement using the kinematic model (47, 48). We will base our model on an existing model that has been used by our team in a movement analysis study performed on seamstresses (47). Twenty-nine spherical reflecting markers (4 mm in diameter) will be placed on anatomical landmark of upper limbs and thorax. Markers placements are visible in Figure 4 and details of the markers disposition are available in Supplementary material 1. For practical reasons related to the use of laparoscopic forceps and the limited fine motor skills in the fingers during laparoscopy, we will eliminate the distal markers located at the hands. Before each motion capture, we will perform a capture in a neutral posture, the arms slightly in abduction with a slight internal rotation at the shoulders (abduction <30°) (49). Ranges of motion of the different degrees of freedom and CMC will be extracted.

### Data analysis and management

#### Sample size

The sample size will be reach based on the availability of the OBGY residents and surgeons in our department and their availability during work hours. We estimate the sample size to reach 30 participants. Previous literature had shown similar sample sizes various between 5 and 20 (34, 35, 45). Such sample size (30 participants) base on time resource constraint (50), according to GPower 3.1 software calculation, allows detecting a moderate effect size of *f* = 0.30 with a power of 0.8 for the Wilcoxon signed-rank test with a level of significance of 5%.

#### Data management

The recruiting clinicians will keep a register with a study number and all identifiable data (name, phone number, mail, pseudonymization code) for use. The “data will be stored in a locked cabinet in the department of gynecology accessible only by the main investigator (ASe). The digital data will be saved on an external hard drive. Data will be stored for a period of 10 years after the publication of the results.

#### Statistical analysis

The level of significance for all statistical analyses will be set at 0.05 under the bilateral hypothesis.

Our primary outcome represented by the time to complete the SUTT task will be analyzed using the Wilcoxon signed-rank test.

The normality of quantitative data will be assessed using a graphical method and a Shapiro test. The dimensional consistency of the subjective data will be calculated using Cronbach’s alpha coefficient. We employed a Type III ANOVA to analyze the EMG data, assessing the significant effects of both experimental sessions (i.e., control versus fatigue) while accounting for potential variations between muscles, thereby ensuring a robust evaluation of the factors influencing muscle activation patterns.

To study our secondary outcomes with the force platform, we will conduct a multivariate analysis of variance (ANOVA) as a protected procedure to test the statistical dependence of the COP excursions, maximum COP velocities.

In EMG’s we will conduct an ANOVA to test the statistical dependance of RMS and MPF.

In kinematics, to compare CMC and range of between the two sessions, a one-way ANOVA with post-hoc (Bonferroni) will be performed for each DoF.

All statistical tests will be performed using Jamovi version 2.3.28.

#### Subgroup analysis

We choose to perform a subgroup analysis on experience, expecting a raise of the impact of fatigue in younger trainees in muscular fatigue (35) or in mental fatigue (22). We choose to dived participants into two experience categories such as: Experienced surgeons (post graduated surgeons) and Trainees (Resident).

#### Data monitoring and quality assurance

The process of the study will be monitored by the principal investigator (ASe). The occurrence of adverse event due to fatigue will be monitored. Our intervention is taking place in standard work hours therefor the occurrence of adverse event is small and will not be included as a primary or secondary outcome.

#### Trial status

Recruitment started in March 2023 and ended in September 2023. Data collection is planned to last until May 2024 and analysis until September 2024. The strength of our study lies in the exhaustiveness of the data collected. To date, there is no study in the literature which analyzes EMG, force platform and kinematics at the same time. This has necessitated precise writing of the protocol and parameters studied, hence the delay between the start of inclusions and our submission, but no analysis has yet begun, pending publication of the protocol.
