# Supplementary material for: Effect of whole-day work on surgical performance during simulated laparoscopic surgery: study protocol for a controlled cross over laboratory trial
Source: Front Public Health. 2024 Nov 14;12:1423366. doi: 10.3389/fpubh.2024.1423366 (PMC11602330; doi:10.3389/fpubh.2024.1423366)
Supplement: Supplementary file 2 [file Table_2.DOCX]

| **STUDY PERIOD** | | | | | |
| --- | --- | --- | --- | --- | --- |
| **Timepoint** | **Enrolment** | **Allocation** | **Post-allocation** | | **Close-out** |
|  | March 2023 | April 2023 | May 2023 | June 2023 | June 2023 |
| **Enrolment :** |  |  |  |  |  |
| Eligibility screen | X |  |  |  |  |
| Informed consent | X |  |  |  |  |
| One hour training session |  | X |  |  |  |
| Allocation |  | X |  |  |  |
| **Sessions :** |  |  |  |  |  |
| Control : In the Morning |  |  | X | X |  |
| Fatigue: After a workday |  |  | X | X |  |
| **Assessments**  Participants characteristics | X |  |  |  |  |
| Performance |  |  | X | X |  |
| Muscle activation |  |  | X | X |  |
| Muscle fatigue |  |  | X | X |  |
| Force platform |  |  | X | X |  |
| Kinematics |  |  | X | X |  |
| Perceived workload |  |  | X | X |  |
| Motivation |  |  | X | X |  |
| Physical strain |  |  | X | X |  |
| Physical fatigue |  |  | X | X |  |
| Effort |  |  | X | X |  |
