# Supplementary material for: Effect of whole-day work on surgical performance during simulated laparoscopic surgery: study protocol for a controlled cross over laboratory trial
Source: Front Public Health. 2024 Nov 14;12:1423366. doi: 10.3389/fpubh.2024.1423366 (PMC11602330; doi:10.3389/fpubh.2024.1423366)
Supplement: Supplementary file 3 [file Table_3.DOCX]

Supplements

**NASA-TLX:** Anchors words for each dimension

1. Mental Demand

- Low: Little thought or concentration required.

- High: Requires intense thought and maximum concentration.

2. Physical Demand

- Low: No physical demands or minimal effort required.

- High: Very intense and demanding physical effort.

3. Temporal Demand

- Low: Plenty of time available to complete the task.

- High: Very limited time, strong time pressure.

4. Performance

- Low: Very unsatisfactory performance, unable to complete the task.

- High: Excellent performance, fully accomplished the task.

5. Effort

- Low: Minimal effort to complete the task.

- High: Maximum effort required, nearly impossible to sustain.

6. Frustration

- Low: No frustration felt, very enjoyable task.

- High: Very high frustration, significant difficulty in completing the task.

**SURG-TLX:** Anchors words for each dimension

1. Mental Demands

- Low: Low mental demand, no cognitive pressure.

- High: High mental demand, requiring constant attention.

2. Physical Demands

- Low: No physical demands, easy task to perform.

- High: Very physically demanding task, significant fatigue.

3. Temporal Demands

- Low: No time constraints, ample time to think.

- High: Intense time pressure, very short deadlines.

4. Task Complexity

- Low: Simple and easy-to-understand task.

- High: Complex task, requiring great analytical ability.

5. Situational Stress

- Low: Calm and relaxed environment, little stress.

- High: Stressful environment, high emotional pressure.

6. Distraction

- Low: No distractions, full concentration.

- High: Many distractions, difficulty concentrating.
